# Diversity from similarity: cellular strategies for assigning particular identities to actin filaments and networks

**DOI:** 10.1098/rsob.200157

**Published:** 2020-09-02

**Authors:** Micaela Boiero Sanders, Adrien Antkowiak, Alphée Michelot

**Affiliations:** Aix Marseille University, CNRS, IBDM, Turing Centre for Living Systems, Marseille, France

**Keywords:** actin isoforms, tropomyosin, post-translational modifications, network architecture, actin-binding protein segregation, actin filament identity

## Abstract

The actin cytoskeleton has the particularity of being assembled into many functionally distinct filamentous networks from a common reservoir of monomeric actin. Each of these networks has its own geometrical, dynamical and mechanical properties, because they are capable of recruiting specific families of actin-binding proteins (ABPs), while excluding the others. This review discusses our current understanding of the underlying molecular mechanisms that cells have developed over the course of evolution to segregate ABPs to appropriate actin networks. Segregation of ABPs requires the ability to distinguish actin networks as different substrates for ABPs, which is regulated in three different ways: (1) by the geometrical organization of actin filaments within networks, which promotes or inhibits the accumulation of ABPs; (2) by the identity of the networks' filaments, which results from the decoration of actin filaments with additional proteins such as tropomyosin, from the use of different actin isoforms or from covalent modifications of actin; (3) by the existence of collaborative or competitive binding to actin filaments between two or multiple ABPs. This review highlights that all these effects need to be taken into account to understand the proper localization of ABPs in cells, and discusses what remains to be understood in this field of research.

## 1. Introduction

Actin plays a major role in many different biological processes such as cytokinesis, migration, vesicular trafficking and infection [1,2]. For each of these functions, actin filaments are organized into networks of optimized architectures, dynamics and mechanical properties. The main types of organizations include (but are not limited to) branched and linear networks of actin filaments [3,4]. Branched actin networks are generated by the association of a seven-subunit complex called the Arp2/3 complex, which nucleates short actin filament branches. Linear networks, where polar actin filaments are parallel or randomly organized, are generated from the de novo nucleation of actin filaments by factors such as formins, or from the debranching and reorganization of branched networks.

Actin networks are regulated by the association of different families of actin-binding proteins (ABPs). It is important to note that although all these proteins coexist in the cell cytoplasm, only a specific subset of ABPs interacts with each actin network while being excluded from the others [5–7]. Such an observation is surprising since it would be natural to assume that all actin filaments in the cell represent equivalent substrates for ABPs. On the contrary, these observations reveal the existence of complex mechanisms capable of precisely addressing the cell's ABPs, and research conducted in recent years has revealed a much more complex picture than anticipated. This work will review the multifarious strategies that cells use to guide ABPs to the appropriate actin networks, the molecular mechanisms behind these processes, and discuss future directions for research in this area.

## 2. Cellular strategies for distinguishing actin networks as different substrates for actin-binding protein binding

There are multiple arguments to assert that the binding of most ABPs to specific actin subnetworks does not rely solely on their transport or on their local activation. First of all, actin networks in cells are often very close to each other. Sometimes an actin subnetwork can even form from a pre-existing one. This is the case, for example, of filopodia, which emerge through the elongation of actin filaments assembled in lamellipodia. In this context, the fast diffusion of proteins in the cytoplasm of cells (typical diffusion rates measured for globular proteins of 10 to 100 kDa range from around 10 to 100 µm^2^ s^−1^) would prevent a precise and efficient targeting of ABPs [8,9]. Second, further evidence comes from the fact that local activation of specific actin assembly pathways in cells is often sufficient to induce the formation of functional actin networks. For example, the recruitment by optogenetic tools of specific RhoGTPases is sufficient to trigger actin assembly, and to initiate actin-dependent processes such as cell migration [10] or cytokinesis [11]. Similarly, triggering actin assembly from cellular extracts by specific factors such as WASp (which is an activator of the Arp2/3 complex) or formins, leads to the formation of actin filament networks with a composition of ABPs comparable to branched and linear actin networks, respectively [12–14].

**Figure 1.**
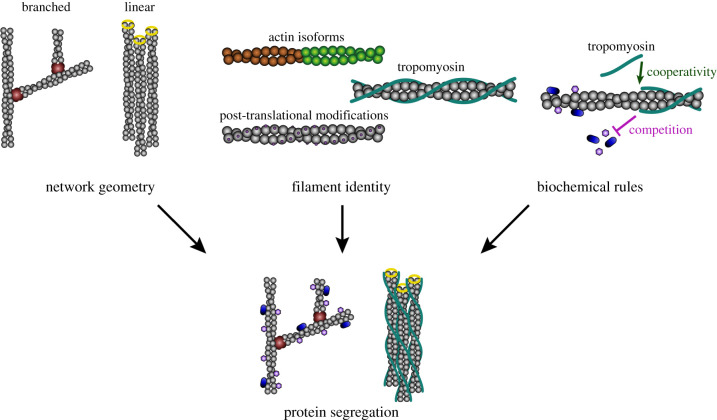
Three main mechanisms that account for the segregation of ABPs to different actin networks in cells. Schematic of the different molecular mechanisms described in this review. Actin networks are distinguished by different geometries. For example, the Arp2/3 complex generates branched networks and formins generate linear arrays. Actin filaments have different molecular identities based on the use of various actin isoforms, post-translational modifications and/or the presence of tropomyosin. Additionally, ABPs can compete or cooperate to restrict or promote their binding to actin filaments.

All of these observations unambiguously indicate that, to a large extent, actin networks themselves represent different substrates for downstream protein interactions. This has led the community to ask what specific features could allow actin networks to distinguish themselves from each other, in order to be identified as different substrates for the cell's ABPs [15]. To date, two main hypotheses, not mutually exclusive, are guiding this field towards a better understanding of these principles. The first hypothesis is that the geometrical organization of filaments within actin networks is itself a sufficient characteristic to make these substrates distinct for ABPs (figure 1). The second hypothesis is that the actin filaments themselves within the actin networks could present different biochemical signatures, which could differentiate them for the different ABPs of the cell (figure 1).

## 3. The geometry of actin networks as an intrinsic feature of actin-binding proteins segregation

Actin filaments that are polymerized *in vitro* for standard actin-binding assays are generally between 1 and 100 µm in length, corresponding to approximately 360–36 000 actin subunits [16]. Since actin filaments are semi-flexible polymers with a persistence length of about 17 µm, this size range is of interest for studying certain biophysical aspects of actin filaments and their interactions with ABPs [17]. However, since most actin filaments *in vivo* are shorter than 1 µm, the results of these studies may bias our interpretation of how ABPs interact with actin filaments in cells.

Furthermore, actin filaments also differ significantly from one actin network to another. Primarily, they vary in length and relative orientation to each other. For example, the branched actin networks of the lamellipodium are composed of short actin filaments of 7–18 actin subunits [18], which are connected to each other by the Arp2/3 complex at an angle of 70°. At endocytic sites, filaments are also branched but appear to be longer, between 18 and 68 actin subunits [19–22]. At these scales, actin filaments are short enough to be considered totally rigid. Linear arrays, on the contrary, are often composed of longer filaments with up to 300 actin subunits [23,24]. The actin filaments are approximately parallel to each other, and may all have similar (e.g. in the case of filopodia) or random (e.g. in the case of cytokinetic rings or stress fibres) orientations.

**Figure 2.**
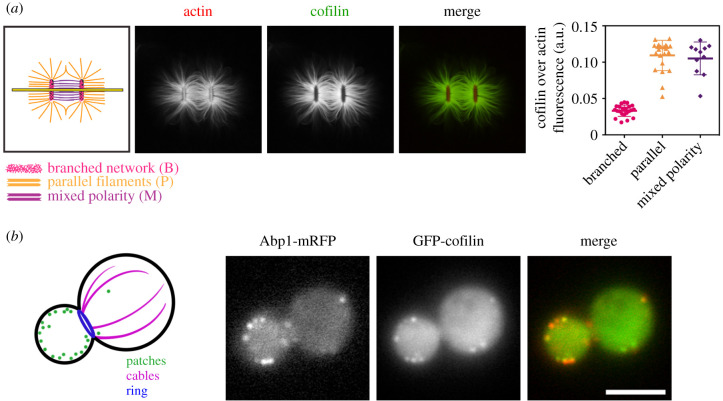
Actin network architecture as an important property to consider in explaining the accumulation of ABPs. (*a*) Profile of accumulation of ADF/cofilin on different actin network architectures reconstituted *in vitro* (adapted from [36]). Left: Schematic of the different actin network architectures assembled on a micropattern coated with an activator of the Arp2/3 complex (two vertical bars), in the presence of soluble actin and Arp2/3. Branched actin networks are assembled on the patterns, whereas linear actin networks (parallel or mixed polarity) are assembled from the elongation of the filaments away from the patterns. Middle: Localization of ADF/cofilin (in green) after its addition to polymerized actin network. Cofilin accumulates preferentially to linear actin networks. Right: Quantification of ADF/cofilin over actin intensities for each architecture. (*b*) Accumulation of ADF/cofilin on different actin network architectures *in vivo* (adapted from [38]). Left: Schematic of the different actin networks found in *S. cerevisiae* cells: branched networks in actin patches (dots), linear networks in actin cables (intra-cellular lines) and the cytokinetic ring (at the yeast bud neck). Right: ADF/cofilin (in green) co-localizes preferentially with the actin patch protein Abp1 (in red), indicating that other principles than network architecture are at play to account for the cellular localization of ADF/cofilin.

Studying the relationship between the geometrical organization of actin networks and the apparent affinity of ABPs has required the development of more complex biomimetic systems than previously envisaged. The best description to date of the impact of actin network organization on the binding and activity of an ABP concerns myosins, particularly contractile myosins with multiple motor domains [25,26]. Several studies have shown that their recruitment and activity strongly depend on whether the actin networks are disorganized, branched by the Arp2/3 complex, parallel or antiparallel [27–29]. More particularly, these motors are capable of strong contractile activity, even to the point of disassembling actin networks, when actin filaments do not have the same orientation and polarity. These observations are very consistent with the effects of these molecular motors *in vivo*. This is the case, for example, at the sarcomeres of muscle fibres or at cell–cell junctions in tissues, where the contractile activity of the motors is exerted on actin filaments of opposite polarity [30]. This is also the case for many disorganized actin networks where myosin activity is capable of driving actin flows or pulsatile phenomena [31–33]. On the contrary, actin filament structures such as filopodia, where the actin filaments all have the same orientation, are not contractile structures [4,34]. On these structures, molecular motors are generally used for trafficking. It is important to note that the sensitivity of ABPs to different actin filament organizations does not seem to be limited to the case of molecular motors, but seems, on the contrary, to be quite general. For example, crosslinkers such as α-actinin bind preferentially when the spacing between two actin filaments is favourable [35]. Other proteins such as ADF/cofilin, which is involved in the disassembly of actin networks, accumulate on linear networks of actin filaments, but are not efficiently recruited on branched actin networks *in vitro* [36] (figure 2*a*).

The geometrical organization of actin networks is, therefore, a key parameter to consider when evaluating the affinity and activity of ABPs. However, other observations made at the cellular level show that the geometric organization of actin networks alone is not sufficient to fully address how ABP recruitment is carried out *in vivo*. Indeed, some proteins, such as ADF/cofilin mentioned above, are not found in cells on actin networks for which their affinity should be the strongest. While ADF/cofilin is clearly localized on branched actin networks such as endocytic actin patches, ADF/cofilin is hardly detectable on linear networks such as actin cables [37–39] (figure 2*b*). These observations indicate that additional principles, beyond the actin filament network architecture, need to be taken into account to obtain a global picture of how ABPs are addressed in cells.

## 4. Biochemical opportunities for generating different actin substrates

Although attractive, we saw that the segregation of ABPs observed in cells could not be explained solely by the geometric organization of actin filaments. The necessity to discriminate different populations of actin filaments in cells must lead us to consider other possibilities, including that small differences in structure and surface properties of the actin filaments themselves could modulate their affinity for certain ABPs. Our community has been working on a number of additional hypotheses to explain how actin filaments could be functionally different and acquire identities of their own. A first hypothesis is that cells could possibly use different actins, either through the expression of different actin isoforms or through post-translational modifications (PTMs) [40,41]. A second hypothesis is that specific ABPs could progressively decorate actin filaments to give them a specific identity, reinforcing or limiting the binding of other ABPs to the same filaments by steric effects or by stabilizing particular conformations of the filaments [5]. These two hypotheses are not mutually exclusive, meaning that cells could also use several strategies simultaneously to create the greatest possible diversity of actin substrates.

**Figure 3.**
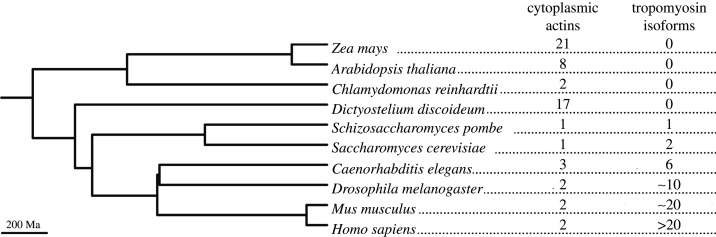
Phylogenetic tree showing how the number of cytoplasmic actin and tropomyosin isoforms changed over the course of evolution in eukaryotes. These numbers are strongly anti-correlated, suggesting that different lineages used different strategies to generate a complex actin cytoskeleton. For clarity, muscle actins which are specific to Metazoa and which are highly specialized were excluded.

Proof that both of these strategies exist in cells is evident from a careful analysis of genomes of all eukaryotes (figure 3). Many species express a variable number of actin isoforms, which can be either cell-specific or expressed simultaneously in the same cell types. An extreme example is plants, which express multiple actin isoforms, that originated from genome duplications (figure 3). The number of actins varies for each plant species and can reach, for example, 21 isoforms in *Zea mays*. A potential limitation of this strategy is that the actin sequence must remain highly conserved in order to maintain its assembly properties and its ability to interact with the most essential ABPs. For example, even between two distant eukaryotes such as budding yeast and human, which diverged more than a billion years ago, actin sequences still retain around 90% identity. Actin mutations are generally rare, and usually lead in humans to serious diseases such as Baraitser–Winter syndrome [42]. Overall, while the existence of many actins in eukaryotes supports the possibility that cells can use a variety of actins to generate different actin-related functions, the difficulty of bringing mutations and generating variety also questions the effectiveness of such a mechanism to generate diversity of functions.

An alternative strategy to differentiate actin filaments without the need to mutate the actin itself is through the use of specialized ABPs. This hypothesis has gained considerable credibility with the comparison of eukaryotic genomes. Indeed, whereas some species, such as plants and amoebas, generally express dozens of different actin isoforms, other eukaryotes, for example, those from the kingdoms Animalia or Fungi, express only one or a very limited number of cytoplasmic actin isoforms (figure 3). Conversely, species expressing one or few actin isoforms express a multitude of tropomyosins, which are specific ABPs that wrap around actin filaments, whereas plants or amoebas do not express any (figure 3) [43]. This very strong anticorrelation is a signature that these two phenomena are probably related to each other. It suggests that while some species use multiple actin isoforms, representing different substrates for ABPs, in order to create different actin-related functions, other species that had gained tropomyosins in the course of evolution could use a limited number of actins, decorated by different tropomyosins, to generate functional diversity.

## 5. Generating a diversity of actin substrates by expressing a variety of actin isoforms

The question that will now be addressed is whether very similar eukaryotic actins are nevertheless able (1) to assemble separately within a common cytoplasm and (2) to form filaments of sufficiently specific molecular identity to interact differently with ABPs and carry specific cellular functions. The answer to this question is far from being clear today and is the subject of intense research.

### 5.1. Plant actins localization and functions

Plant actins were originally studied from the model organism *Arabidopsis thaliana*, which has 10 actin genes. Eight of these genes have been demonstrated to code for functional actin isoforms, grouped in two classes according to their sequence similarities and their tissue-specific expressions: vegetative (ACT2, 7 and 8) and reproductive (ACT1, 3, 4, 11 and 12) [40]. Vegetative and reproductive actins are involved in different cellular processes [44], and plant actin isoforms that are expressed in the same tissue can also assemble into isoform-specific structures. GFP-fusion proteins of ACT2 and ACT7, the main vegetative actin isoforms, co-localize only partially at the surface of chloroplasts, where ACT2 is mainly found in thinner and longer bundles, whereas ACT7 is organized into thick bundles [45]. Besides their differential expression and their spatial segregation, these isoforms are not functionally equivalent. Expression of the reproductive actin ACT1 in vegetative tissues causes dwarfing and altered morphology in most organs, showing that expression of ACT1 in these tissues is affecting the dynamics of actin and its associated proteins [46].

Another well-studied organism is the unicellular green algae *Chlamydomonas reinhardtii*, which carries two actin genes. The main isoform IDA5 is a conventional actin that is expressed in normal conditions. The second actin, called NAP1 (for Novel Actin-like Protein 1), is highly divergent as it shares only 65% sequence identity with IDA5 [47]. The expression of NAP1 in wild-type cells is negligible, but it is highly upregulated in certain conditions, for example, when IDA5 is absent or after addition of the actin monomer sequestering drug latrunculin B [48,49]. This drug can prevent IDA5 polymerization, but surprisingly NAP1 generates latrunculin B-resistant structures. Despite being so different, essential actin functions can be performed by either of these actins, and cells lacking any of the actin genes can grow and divide normally. However, they also seem to maintain some specialized functions. The conventional actin IDA5 has a function in mating since it is involved in the elongation of the fertilization tube, a function that NAP1 cannot substitute [50–52]. Both IDA5 and NAP1 are found in the axoneme of the flagella but apparently in different structures. While IDA5 seems to be part of the inner dynein arms, NAP1 plays a role in flagellar formation independently of axonemal dyneins [52,53].

### 5.2. β- and γ-actin localization and functions

**Figure 4.**
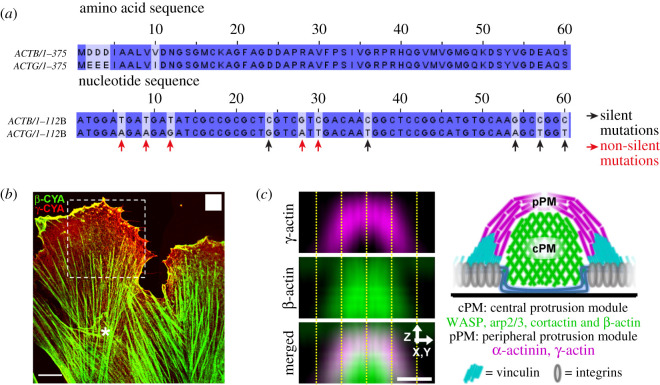
Evidence that highly similar cytoplasmic actins can nevertheless assemble into functionally different actin networks in cells. (*a*) Beginning of the nucleotide and amino acid sequence of β- and γ-actins. These proteins only differ in four amino acids located at the N-terminal end, although their nucleotide sequences have a much higher number of silent mutations (e.g. black arrows). (*b*) Example of the differential localization of β- (green) and γ-(red) actins at the cell scale, in migrating human subcutaneous fibroblasts (adapted from [54], scale bar, 10 µm) (*c*) Another example of the differential localization of β- (green) and γ-actins (magenta) within a same structure, podosomes (adapted from [57], scale bar: 0.5 µm). A linear network of γ-actin is surrounding a branched β-actin core.

Generating actin structures from different actin isoforms is also possible in higher eukaryotes, including mammals. Mammalian organisms have six different actin isoforms: four muscle actins and two cytoplasmic actins. The latter, called β- and γ-actins, differ only in four amino acids at the N-terminal end (figure 4*a*) and are simultaneously expressed in cells. Due to their extreme similarities, determining the cellular localization of β- and γ-actin is challenging. Specific monoclonal antibodies, recognizing specifically the different N-terminal regions, are now available to visualize the localization of both isoforms in different cell types [54,55].

β-actin was originally found mainly in actin bundles of basal stress fibres, filopodia, at cell–cell contacts and in contractile rings, whereas γ-actin is present mainly in lamellar and dorsal cell regions (figure 4*b*) [54,55]. In epithelial cells, β-actin has been shown to play a role in adherens junction maintenance, and γ-actin in tight junction integrity [56]. In podosomes, which are actin-rich adhesive structures involved in migration and invasion, the use of better super-resolution microscopy techniques allowed a differential localization between actin isoforms to be distinguished. Actin filaments in podosomes are organized into two distinct networks, consisting of a β-actin core, composed of branched actin filaments nucleated with WASp, Arp2/3 and cortactin, and surrounded by a γ-actin envelope, composed of linear actin filaments bound to α-actinin and connected with myosins (figure 4*c*) [57].

These differences in localization suggest that these two isoforms, despite being so similar, could be associated with different cellular functions. During wound closure, cells assemble significantly more β-actin beneath the plasma membrane, which suggests a role for this actin in cell motility [58]. β-actin implication in cell motility was confirmed in fibroblasts where decreased β-actin protein levels lead to reduced motility [59,60]. The implication of γ-actin in cell migration is less clear. While γ-actin knocked-down cells are shown to migrate less in some studies [54,61], loss of γ-actin can also induce epithelial-to-mesenchymal transitions in another model [62]. In agreement with β-actin localization in the cytokinetic ring, β-actin knocked-down cells also show reduced proliferation and can be multinucleated [59,60,63,64]. In breast cancer cells, cycle entry and proliferation seem to be regulated by γ-actin, particularly in G1, while β-actin plays a role in later mitotic stages, especially in telophase for cytokinesis [64]. β-Actin implication in cell motility and proliferation can be explained by the direct activity of β-actin in the filaments of the structures controlling these processes, but also by the role that this specific actin plays in the regulation of transcription. β-Actin binds directly to chromatin remodelling proteins as well as RNA polymerases, a first indication of its role in nuclear processes [65]. This role is also confirmed as β-actin was shown to regulate the expression of cell cycle and actin dynamics related genes, as well as its own expression [59,66].

Cellular localization of these actins does not suggest any particular preference for a certain type of architecture. For instance, β-actin is localized at the linear structure of the stress fibres and contractile ring but it is also localized at the branched core of the podosomes. This lack of a general obvious rule complicates our understanding of the molecular mechanisms implicated in the assembly of these two actin isoforms into distinct networks. Moreover, this understanding is further complicated by functional tests at the whole organism level. Despite their amino acid sequences being so similar, the nucleotide sequences of beta and γ-actin genes possess silent mutations that affect 40% of the codons (figure 4*a*). By taking the β-actin gene, and changing only four codons to express γ-actin from this gene, it is possible to generate viable mice that are not expressing the β-actin protein [67]. This result is surprising, since β-actin knock-out mice are reported to be embryonic lethal [68,69]. Therefore, essential functions of β-actin may not be related primarily to its amino acid sequence, but may also rely heavily on its nucleotide sequence. This difference in nucleotide sequence results in different translation speeds [70], which could lead to protein regulation at different levels: differential expression levels, alternative splicing and differential co- and PTMs.

### 5.3. Biochemical similarities and differences between actins

Differences in cell localization described above suggest that different actins, although very similar, must still have significantly different biochemical properties. However, while divergent actins expressed by prokaryotes have clearly distinguishable assembly properties, actins expressed in eukaryotes seem to have much more subtle biochemical differences [71,72]. The search for these subtleties has long suffered from the difficulty of purifying a variety of actin isoforms in order to study them independently. Most of our knowledge is based on studies using the same mammalian actin muscle isoform. A more limited number of studies have used the yeast actin *S. cerevisiae*, and only a few studies used mixtures of γ- and β-actin or actins from other species.

Actins from budding yeast and rabbit muscle are 87% identical, which indicates that these two actins are quite different comparatively to all actins expressed in eukaryotes. Budding yeast and rabbit muscle actins can nevertheless copolymerize [73]. Surprisingly, this is less clear for beta and γ-actin, despite being 99% identical. While these two isoforms were shown to copolymerize in some studies, other studies reported their ability to assemble into independent filaments [55,74,75]. Yeast and rabbit muscle actins show differences in flexibility, with a persistence length of rabbit muscle actin two-to-threefold higher than yeast actin [76,77]. In the presence of magnesium, yeast actin polymerizes faster than muscle actin, which is due to a faster trimer nucleus formation rather than a faster elongation of the filaments [78–81]. This difference in rates of polymerization is also observed in plants, as the two vegetative actins ACT2 and ACT7 polymerize faster than the reproductive actins ACT1 and ACT11 [82]. Nucleotide hydrolysis, nucleotide exchange and Pi release are also faster for yeast actin compared to muscle actin filaments [80,81,83–85]. This correlates with the fact that the nucleotide-binding cleft of *S. cerevisiae's* actin appears more open than for muscle actin [86]. In summary, we can hypothesize from few well-characterized actins, that many biochemical and biophysical subtleties might overall account for important functional differences in cells.

Differences among actins are also sufficient to modulate some interactions with ABPs. Actin nucleators, which play an important role in architecture formation, are reported in few studies to favour specific actin isoforms. The formin DIAPH3, for example, has a preference for β-actin compared to γ-actin, suggesting that actin cables assembled from DIAPH3 could be enriched with β-actin [55]. The VCA domain of N-WASP, an activator of Arp2/3, does not show specificity for β- or γ-actin [87], but *S. pombe*'s Arp2/3 is reported to be a better nucleator of *S. pombe*'s actin than rabbit muscle actin [80]. In *Arabidopsis thaliana*, the binding affinity of profilins for actin monomers seems lower for a specific isoform, ACT2 [82]. Since profilin enhances formin-linear actin cable assembly, at the expense of Arp2/3-branched network assembly, it is tempting to speculate whether ACT2 would assemble more specifically within branched networks. Moreover, to achieve different actin functions, it appears that plant actins and ABPs have co-evolved to generate class-specific protein–protein interactions. The expression of the reproductive ACT1 isoform in vegetative tissues leads to aberrant cell and tissue morphology, a phenotype that is rescued by co-expression of the reproductive profilin (PRF4) and cofilin (ADF7) [88]. Evidence for coevolution of actin with ABPs can be also found by studying proteins from different species. For example, in both yeasts *S. cerevisiae* and *S. pombe*, profilin inhibits endogenous actin polymerization but has little effect on rabbit muscle actin polymerization [81,89,90]. Another example is vertebrate cofilin, which can bind to *S. cerevisiae's* actin but does not increase their flexibility nor promote severing [77,91].

These studies indicate to the scientific community that highly similar actin isoforms have subtle but significantly different properties to display preferential binding to a variety of ABPs. We are just beginning to identify the molecular mechanisms by which actin isoforms could assemble into distinct actin networks of specialized properties. However, we still do not have a satisfying overview of the variety of possible differences among all actin isoforms expressed in eukaryotes. Recently, new protocols have been developed [92–94], allowing for a wider variety of actin isoforms to be purified. It is likely that future comparisons of a greater diversity of actin isoforms, purified from similar protocols, will strengthen our knowledge of these mechanisms.

## 6. Actin's post-translational modifications

Co- and PTMs are covalent modifications to one or several amino acids of a protein, a process that is usually mediated by specific enzymes. These modifications can affect the interactions of the protein with its partners by changing its surface charge density, its structure, or by steric hindrance.

First, actin can be arginylated, which is the addition of an arginine residue at the N-terminal end. This modification is mediated by the arginyl-tRNA-protein transferase Ate1, a protein that has been identified in several organisms, including mammals, plants and budding yeast [95]. In *Dictostellium discoideum*, an organism expressing a large number of cytoplasmic actin isoforms, several actins (Act3, Act10, Act17, Act22, Act23 and the most abundant one Act8) are arginylated, and impairing Ate1 activity affects cell migration and substrate adhesion [96]. In mammals, arginylation is possible for β- and γ-actins, but the latter is specifically degraded when it is arginylated [70,97]. As this PTM does not affect both actin isoforms equally, arginylation could be an important PTM to regulate specifically β-actin-dependent cellular processes. For example, arginylated β-actin, which corresponds to around 1% of total β-actin, is likely to be involved in lamella formation, as downregulation of Ate1 reduces the formation of this structure [97–99].

Studies in Ate1 knocked-out cells indicate that actin arginylation is responsible for a decreased interaction with gelsolin, but for an increased recruitment of capping protein (CP) and twinfilin [100]. Arginylation adds positive charges to normally negative charged surfaces, a change that logically affects actin's interaction with binding partners such as gelsolin, whose binding relies on the first 10 amino acids of actin [101,102]. On the contrary, CP and twinfilin are not shown to bind to this area, but their increased binding could be explained by an absence of gelsolin which would leave excessive free actin filament barbed ends for these two proteins to bind to.

The most abundant PTM for β- and γ-actin is N-terminal acetylation, which is the addition of an acetyl group [103]. In animals, this PTM occurs after cleavage of the first one or two amino acids, and is modifying an important fraction of the actin [104–107]. It is mediated by the acetyltransferase NAA80, which is specific to actin and acetylates preferentially the monomeric actin-profilin complex [108,109]. Interestingly, plants and fungi do not express NAA80, but do express the general acetylase NatB, which acetylates many other proteins. In yeast, actin is co-translationally acetylated by NatB [110,111], but in plants, the role of NatB is less clear. Even though the lack of NatB affects plant growth, actin is not identified as a substrate for this protein [112]. As NatB targets the N-terminal part of proteins starting with Met-Glu-, Met-Asp-, or Met-Asn-, plant actins, which start with Met-Ala-, may not be modified or may be modified by a mechanism not yet identified [111]. Since plants like *Arabidopsis thaliana* already express several actin isoforms, we can also speculate that actin acetylation might be less important to generate different actin-based functions in this organism. In HeLa cells, actin acetylation affects cell motility and cytoskeletal organization [103]. Acetylated actin has a faster polymerization rate, including formin-induced polymerization, and a faster depolymerization rate, so filaments composed of acetylated actin are shorter lived [103]. The N-terminal residue of actin is not the only amino acid that can be acetylated. A complex of lysine-acetylated actin and cyclase-associated protein (CAP) was shown to promote the inhibition of the formin INF2 [113]. This proves that PTMs can not only regulate actin properties and its binding to other proteins, but also the activity of the other proteins themselves.

Arginylation and acetylation are two main PTMs of actin. Other PTMs, including phosphorylation and methylation, can also modify the chemistry of the actin molecule. For more details, we refer readers to a more detailed review [114].

## 7. Tropomyosins and the biogenesis of new actin substrates

### 7.1. Generating diversity from a limited number of actin isoforms: tropomyosin as the missing link

We will now study the more complex case where a cell is able to generate distinct actin networks from identical (or nearly identical) actin molecules. The distinction between actin networks can no longer be made on the basis of biochemical differences between the actin composing different networks, but on the basis of biochemical particularities of the ABPs composing each of the networks. For greater clarity, our discussion will distinguish two different cases: the first case corresponds to the de novo generation of new actin networks from determined actin nucleation factors; the second case corresponds to the reorganization of pre-existing networks into networks with different properties.

In the first case, the assembly of new actin filament networks suggests that filaments acquire particular identities at the moment when they are generated by nucleation factors. The idea that this function is carried by factors such as the Arp2/3 complex or formins is *a priori* tempting. However, the coincubation of the Arp2/3 complex, its VCA activator and a formin (FMNL2) leads to the formation of mixed actin networks (i.e. having both Arp2/3 branches and formin-bound filaments) and not of distinct actin networks [115]. It should be noted that in the experiment described, the actin filament branches are much longer than the branches present in the cells, and that we could not exclude the possibility that formin cannot bind to very short branches. It is also possible that formin FMNL2 is a peculiar isoform that can bind to branched networks [115,116]. Nevertheless, this experiment rather suggests that another regulator is needed to effectively segregate formins and the Arp2/3 complex on separate networks. We have already seen in paragraph 4 that careful genomic analysis strongly suggests that proteins of the tropomyosin family are responsible for functional diversity of the actin cytoskeleton in higher eukaryotes [43]. We shall see that genetics, cell biology and biochemistry have also provided additional evidence for the importance of tropomyosins.

The second case corresponds to situations where actin networks undergo major dynamic reorganizations, independently of any nucleation of new actin filaments. For instance, linear actin structures found beneath the lamellipodia are not exclusively generated by formins, but also emerge to a large extent from pre-existing lamellipodial actin networks [117–120]. Interestingly, in this case, where actin filaments are not generated de novo, tropomyosin recruitment correlates also very well with filament debranching and the re-organization of actin filaments into new linear actin structures [121,122]. These observations suggest that regardless of the mechanism by which actin networks are formed, tropomyosins are consistently of key importance in giving actin filaments a new identity.

### 7.2. Tropomyosins localization and functions

**Figure 5.**
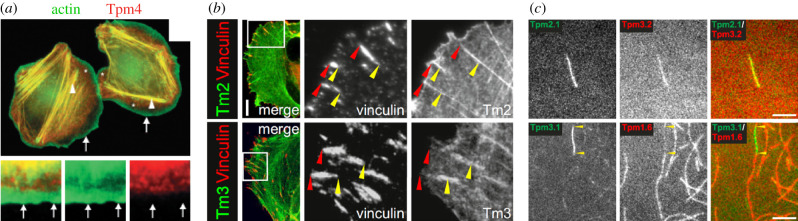
Functional differentiation of actin networks by tropomyosins. (*a*) Example of the specific localization of tropomyosin 4 (in red) in MTLn3 cells. Tropomyosin localizes with actin (in green) in stress fibres and in lamellar structures (arrowheads), while it is absent from the branched actin structures at the leading edge (arrows) (adapted from [128]). (*b*) Example of the differential localization of tropomyosin isoforms (in green) in U2OS cells. While Tpm2 shows a strong colocalization with the focal adhesion-specific protein vinculin (in red), Tpm3 localizes proximally to focal adhesions (adapted from [131]). (*c*) *In vitro* single-filament scale imaging reveals that while some tropomyosin isoforms (top images: Tpm2.1 in green and Tpm3.2 in red) can copolymerize with actin filaments, others cannot (bottom images: Tpm3.1 in green and Tpm1.6 in red) (adapted from [134], scale bar, 5 µm).

Most cells express multiple tropomyosin isoforms and splicing variants, and those proteins have been proposed to provide actin filaments specific identities [5,123–125]. This concept has been reinforced by the observation of cellular localization of tropomyosins, which is highly dependent on the type of tropomyosin isoform [126,127] (figure 5*a*). Among the tens of isoforms that exist in metazoans, individual actin structures usually interact with a subset of tropomyosins. Most actin networks in cells are decorated by specific families of tropomyosins, including filopodia, lamella and stress fibres, with the exception of branched networks such as lamellipodia or endocytic actin patches which do not recruit tropomyosins (figure 5*b*) [124,125,127,128]. Some tropomyosins do not form copolymers (figure 5*c*), indicating that they are therefore involved in many different cellular functions [126,129], and modulation of the expression of tropomyosins triggers specific cellular responses. For example, some cancer cell lines can remarkably recover rigidity sensing and rigidity-dependent growth, when a single tropomyosin isoform (Tpm2.1) is over-expressed [127,130]. However, structures such as stress fibres are highly sensitive to the expression level of any isoform of tropomyosin [127,131]. Recent data also suggest that modulation of any tropomyosin isoform impacts the whole myosin organization in cells, thus acting on both tension and traction forces driven by focal adhesions [132]. Therefore, some actin networks might bind to multiple families of tropomyosins simultaneously, which is coherent with the high concentration of tropomyosin present in cells, and with the fact that some tropomyosin isoforms have the ability to copolymerize as demonstrated *in vitro* (figure 5*c*) [133,134].

The presence of tropomyosins on linear actin networks suggests a preference for formin-generated filaments. Indeed, incubation of the pan-formin inhibitor SMIFH2 in oocytes decreases the cortical tropomyosin level [135]. Tropomyosin depletion promotes the expansion of lamellipodia while its overexpression inhibits this branched structure while promoting linear networks [136–138]. Moreover, it seems that some formins assemble actin filaments bound to specific tropomyosins. This is beautifully illustrated in fission yeast, where an exchange of the localization of the fission yeast formins For3 and Cdc12 results in an exchange in localizations of the tropomyosin forms on the corresponding actin networks [139]. Also, the absence of CP in fission yeast cells induces simultaneously ectopic recruitment of the tropomyosin Cdc8 and of both formins Fus1 and Cdc12 [140]. However, specific downregulation of some formins (mDia1 and mDia3) does not affect the localization of tropomyosins, indicating that some formins may not share this specificity for tropomyosins [141].

### 7.3. Impact of tropomyosins on actin filament nucleation, debranching and the binding of other actin-binding proteins

Tropomyosins are dimers of α-helices forming parallel coiled-coils that span several actin subunits [123]. A biochemical link between formins and tropomyosins has been described *in vitro*, and cooperativity between these proteins is established [136,142,143]. In budding yeast, the presence of tropomyosin can specifically increase the nucleation rate of a formin. Conversely, tropomyosins are generally strong inhibitors of Arp2/3-induced actin nucleation and branch formation [144,145]. Debranching and re-organization of actin networks into linear arrays is also favourable to tropomyosins, as this process generates more actin pointed ends from where tropomyosins can bind [121,122]. These observations agree well with the localization of tropomyosin in cells.

The binding of tropomyosin around actin filaments contributes directly to the recruitment of particular families of ABPs, and the dissociation of others. Tropomyosins regulate the activity of the different families of myosins by modifying their binding to actin filaments and their enzymatic kinetics [146,147]. This mechanism is important because it allows cargoes to be directed to appropriate locations and regulates contractility. Elegant *in vitro* studies confirm at the level of single actin filaments that tropomyosin excludes other ABPs, such as fimbrin or ADF/cofilin, therefore preventing filaments from disassembly [129,134,148,149]. Interestingly, tropomyosin is not required *per se* to assemble cables *in vitro* in the absence of disassembling factors but it becomes necessary to maintain cable assembly in biomimetic assays where treadmilling has been reconstituted [136,150]. Tropomyosins are hence major biochemical regulators that define the identity of actin filaments and regulate the binding of many families of ABPs, thereby leading to the segregation of these proteins to different actin networks.

## 8. Regulation of actin networks protein composition by competition between ABPs

Numerous lines of evidence indicate that not only tropomyosins, but most ABPs, display cooperative or competitive binding effects to actin filaments, and that these effects need to be taken into account to understand globally how an appropriate ABP composition of actin networks is reached [5]. A number of cellular biology studies demonstrate unambiguously that the removal of a given ABP from one actin network may trigger a global relocation of ABPs from other actin networks [6,140]. As a consequence, phenotypes observed in cells are not only due to the absence of the ABP of interest, but also to the mislocalization of other ABPs. Several hypotheses could explain this phenomenon. First, it is possible that the absence of a protein in a network may open a binding site for other proteins, or allow the binding of competing proteins. For instance, in yeast, removal of fimbrin from actin patches, which are Arp2/3-branched networks, leads to an ectopic localization of tropomyosin to those networks [6]. Second, it is possible that ectopic protein localization triggers the cooperative binding of additional proteins. For instance, loss of CP from actin patches creates free actin filament barbed ends, where formins can bind, which in turn favours the ectopic binding of tropomyosin [140]. Finally, the absence of an ABP could also have consequences on the geometry of the network, which would consequently impact its ABP composition. Overall, these results indicate that although tropomyosins are key regulators for addressing ABPs to appropriate networks, proper segregation of ABPs on specific actin networks in cells also relies on a global and complex biochemical equilibrium, involving many different families of ABPs. Addressing these questions in the future will require to integrate all these parameters into a comprehensive model.

## 9. Conclusion

The aim of this review was to describe our current knowledge of the different molecular mechanisms involved in the definition of the identity of actin filaments and networks for a proper segregation of ABPs in cells. We conclude this work by emphasizing that these mechanisms are often not purely distinct from each other, but interrelated. A clear example is the fact that a protein like tropomyosin gives an identity to actin filaments, but is also involved in competitive binding with other ABPs. As many different protein–protein interactions and molecular mechanisms are simultaneously involved, a comprehensive understanding of these complex systems requires non-superficial analysis.

## Data accessibility

This article has no additional data.
